# Application of variance components estimation to calibrate geoid error models

**DOI:** 10.1186/s40064-015-1210-5

**Published:** 2015-08-20

**Authors:** Dong-Mei Guo, Hou-Ze Xu

**Affiliations:** State Key Laboratory of Geodesy and Earth’s Dynamics, Institute of Geodesy and Geophysics, The Chinese Academy of Science, 340 Xudong Street, Wuhan, China

**Keywords:** Geoid refining, Weighted least-squares adjustment, Variance component estimation, Errors-invariables model

## Abstract

The method of using Global Positioning System-leveling data to obtain orthometric heights has been well studied. A simple formulation for the weighted least squares problem has been presented in an earlier work. This formulation allows one directly employing the errors-in-variables models which completely descript the covariance matrices of the observables. However, an important question that what accuracy level can be achieved has not yet to be satisfactorily solved by this traditional formulation. One of the main reasons for this is the incorrectness of the stochastic models in the adjustment, which in turn allows improving the stochastic models of measurement noises. Therefore the issue of determining the stochastic modeling of observables in the combined adjustment with heterogeneous height types will be a main focus point in this paper. Firstly, the well-known method of variance component estimation is employed to calibrate the errors of heterogeneous height data in a combined least square adjustment of ellipsoidal, orthometric and gravimetric geoid. Specifically, the iterative algorithms of minimum norm quadratic unbiased estimation are used to estimate the variance components for each of heterogeneous observations. Secondly, two different statistical models are presented to illustrate the theory. The first method directly uses the errors-in-variables as a priori covariance matrices and the second method analyzes the biases of variance components and then proposes bias-corrected variance component estimators. Several numerical test results show the capability and effectiveness of the variance components estimation procedure in combined adjustment for calibrating geoid error model.

## Background

For many years Global Positioning System (GPS) observations and leveling data have been adopted to evaluate gravimetric geoid, and many studies have been carried out by comparing the interpolated values of the gravimetric geoid at the GPS benchmarks with the corresponding GPS-leveling heights (Nahavandchi et al. 2004; Guo and Xu 2011a). The fundamental relationship between the ellipsoidal heights obtained from GPS measurements and the orthometric heights obtained from leveling survey and gravimetric geoid data can be written as (Heiskanen and Moritz 1967),

1 $$h - H - N = 0$$

where *h* is ellipsoidal height obtained from GPS measurements, *H* is orthometric height obtained from leveling survey, *N* is gravimetric geoid data calculated from global geopotential models or regional gravimetric geoid. Based on this inherent appeal of the relationship between ellipsoidal, orthometric heights and gravimetric geoid data, if any two of the heights are given, the third can be derived with Eq. (1).

In practical terms, the performance of Eq. (1) is more complex due to the adaptation of the parametric models for the systematic errors (e.g. long-wavelength systematic errors in N, distortions in the orthometric height due to an over constrained adjustment of leveling network, etc.) and the correctness for the stochastic model of the observable noises due to the covariance (CV) matrices for the height types obtained from separate adjustments of the individual height types.

Up until today, the former problem has been a topic of interest and well studied by different kinds of parametric functions to absorb the systematic errors of ellipsoidal, orthometric heights and gravimetric geoidal undulations. Many models have been performed ranging from a simple linear regression to more complicated models, such as polynomial fit, multiquadric function fit, Spline fit method and so on (Sansò and Sideris 2013). Since it is evident that the suitability of the parameter model depends on the density, distribution and quality of height network data, there is no universal model applicable to any situation. Here the method of multi-surface function is used after evaluation and assessment of parametric models in this paper.

As for the latter issue, the problem of stochastic modeling for observables in the combined adjustment of heterogeneous height types has not yet to be satisfactorily solved by this traditional formulation. This problem will be the main focal point of this paper. In order to reach the best unbiased estimators of the unknown parameters, a proper CV matrix of the observables is required. Here the well-known method of variance component estimation (VCE) is employed to the combined least-squares (LS) adjustment in Eq. (1) (Teunissen and Amiri-Simkooei 2008; Amiri-Simkooei 2013; Wensch et al. 2013). There are many reasons for performing VCE. For example, VCE is an effective statistical tool to test the noise level. This method is very flexible and easily understood. It can be conducted to estimate variance and CV components for linear and nonlinear stochastic model. The main idea of this paper is to give a detailed analysis of the combination adjustment and to estimate all errors of the heterogeneous heights data and to develop a method for calibrating the geoid error models using the method of VCE. In practice, the iterative minimum norm quadratic unbiased estimation scheme is implemented via a combined adjustment using existing ellipsoidal, orthometric heights and gravimetric geoid data.

## Implement of MINQUE to the combined LS adjustment

### Combined LS adjustment of GPS-leveling heights and gravimetric geoid data

To establish the LS variance component estimation, a combined adjustment is carried though using the following functional model with orthometric, ellipsoidal heights and gravimetric geoid data. Considering the Gauss–Markov model, the system of observation equation and the solution can be expressed as,

2-a $$l = Ax + \tilde{v}$$

2-b $$E\left\{ {\tilde{v}} \right\} = 0$$

2-c $$E\left\{ {vv^{T} } \right\} = C_{v} = \sigma^{2} Q_{v}$$

where *A* is the *m* × *t* matrix of known coefficients depends on the parametric model (*m* is the number of observational equations, *t* is the number of unknown parameters), *x* is *t* × 1 vector of the unknown parameters depending on the parametric model, $E\left\{ \bullet \right\}$ is the mathematical expectation operator, *Q*_*v*_ is the *m* × *m* CV symmetric cofactor matrix of the observables, *σ* is the variance or CV components, and the observations vector *l* consists of the height ‘misclosure’ at the GPS-leveling benchmark as follows,

3 $$l_{i} = h_{i} - H_{i} - N_{i}$$

Here, for the design matrix *A* corresponding to the parametric model the model of Multi-surface function fit is being used,

4 $$\zeta \left( {x,y} \right) = \sum\limits_{i = 1}^{K} {a_{i} K\left( {\varphi ,\lambda ;\varphi_{i} ,\lambda_{i} } \right)}$$

where $K\left( {\varphi ,\lambda ;\varphi_{i} ,\lambda_{i} } \right) = \sqrt {\left( {\varphi - \varphi_{i} } \right)^{2} + \left( {\lambda - \lambda_{i} } \right)^{2} + \delta }$, *φ* and *λ* are latitude, longitude respectively, and *a*_*i*_ are the coefficients.

$\tilde{v}$ is a (*m* × 1*)* vector of unobservable random error with zero mean, for each of heterogeneous height data types are given by,

5 $$\tilde{v} = v_{h} - v_{H} - v_{N} = B\left[ {v_{h}^{T} v_{H}^{T} \;v_{N}^{T} } \right]^{T}$$

where *B* is the block-structured matrix $B = \left[ {I{-}I{-}I} \right]$, such that *I* is an *m* × *m* unit matrix.

The corresponding CV matrix is described in Eq. (2-c), which can be written as,

6 $$C_{v} = \left[ {\begin{array}{*{20}c} {C_{h} } & 0 & 0 \\ 0 & {C_{H} } & 0 \\ 0 & 0 & {C_{N} } \\ \end{array} } \right] = \left[ {\begin{array}{*{20}c} {\sigma_{h}^{2} Q_{h} } & 0 & 0 \\ 0 & {\sigma_{H}^{2} Q_{H} } & 0 \\ 0 & 0 & {\sigma_{N}^{2} Q_{N} } \\ \end{array} } \right]$$

where *C* is a positive-definite symmetric matrix. Here we assume that there are no correlations between the heterogeneous height types. *Q*(⋅) are known positive cofactor matrices for ellipsoidal, orthometric heights and gravimetric geoid data, and $\sigma_{h}^{2}$, $\sigma_{H}^{2}$, $\sigma_{N}^{2}$ are the corresponding variance components.

The vector of unknown parameters is computed by using LS minimization principle,

7 $$v^{T} Pv = v_{h}^{T} P_{h} V_{h} + v_{H}^{T} P_{H} V_{H} + v_{N}^{T} P_{N} V_{N} = {\text{minimum}}$$

where the corresponding weight matrix *P* associated with the observations take the forms,

8 $$P = \left[ {\begin{array}{*{20}c} {P_{h} } & 0 & 0 \\ 0 & {P_{H} } & 0 \\ 0 & 0 & {P_{N} } \\ \end{array} } \right] = \left[ {\begin{array}{*{20}c} {C_{h}^{ - 1} } & 0 & 0 \\ 0 & {C_{H}^{ - 1} } & 0 \\ 0 & 0 & {C_{N}^{ - 1} } \\ \end{array} } \right]$$

where *P*(⋅) is assumed diagonal with elements which is the reciprocal of the error variances. According to LS adjustment, the unknown parameters and adjusted residuals of observations can be easily solved. The unknown parameters is given as follows,

9 $$\hat{x} = [A^{T} \left( {C_{h} + C_{H} + C_{N} } \right)^{ - 1} A]^{ - 1} A^{T} \left( {C_{h} + C_{H} + C_{N} } \right)^{ - 1} w.$$

The adjusted residuals of the GPS, leveling and gravimetric geoid height observations can be obtained by

10 $$\hat{v} = CB^{T} \left( {BCB^{T} } \right)\left( {w - A\hat{x}} \right)$$

and

11 $$\begin{gathered} \hat{v}_{H} = C_{h} \left( {C_{h} + C_{H} + C_{N} } \right)^{{ - 1}} Mw\; \hfill \\ \hat{v}_{h} = - C_{H} \left( {C_{h} + C_{H} + C_{N} } \right)^{{ - 1}} Mw \hfill \\ \hat{v}_{N} = - C_{N} \left( {C_{h} + C_{H} + C_{N} } \right)^{{ - 1}} Mw \hfill \\ \end{gathered}$$

with

12 $$M = I - A\left( {A^{T} \left( {C_{h} + C_{H} + C_{N} } \right)^{ - 1} A} \right)^{ - 1} A^{T} \left( {C_{h} + C_{H} + C_{N} } \right)^{ - 1} .$$

And the mean square error and the accuracy of parameters estimations are given as follow,

13 $$\hat{\sigma }_{0} = \sqrt {\frac{{v_{h}^{T} C_{h}^{ - 1} v_{h} + v_{H}^{T} C_{H}^{ - 1} v_{H} + v_{N}^{T} C_{N}^{ - 1} v_{N} }}{m - t}}$$

14 $$D_{{\hat{x}}} = \hat{\sigma }_{0} \left( {A^{T} \left( {C_{h} + C_{H} + C_{N} } \right)^{ - 1} A} \right)^{ - 1} .$$

According to this combined LS adjustment approach, the solution can be achieved depending on two issues, one is the appropriateness of the parametric models, which refers to the correction for the data inconsistencies and the systematic errors, and another is the residuals of the height data types which allow for the calibration of data covariance matrices. Since the former problem has been well studied, the main work of next section focus on a description of the implement of VCE schemes to the combined LS height adjustment.

### Application of MINQUE to the Combined LS Adjustment

There are many methods available to implement VCE within the LS adjustment (Helmert 1924; Rao 1970). The first solution was proposed by Helmert. And an independent solution was proposed by Rao (1970) who put forward a method known as minimum norm quadratic unbiased estimation (MINQUE). Assuming the observations are normally distributed the approach of Helmert’s and Rao’s are equivalent. In this paper the MINQUE procedure is employed since this procedure does not require distributional assumptions.

Here the general MINQUE algorithm is modified to estimate the variance components in the combined LS adjustment with heterogeneous observation types,

15 $$S\hat{\theta } = q$$

where $\hat{\theta }$ is a vector composed of the unknown variance components $\sigma_{h}^{2}$, $\sigma_{H}^{2}$ and $\sigma_{N}^{2}$. The matrix *S* is denoted by

16 $$S = \left[ {\begin{array}{*{20}c} {S_{hh} } & {S_{hH} } & {S_{hN} } \\ {S_{Hh} } & {S_{HH} } & {S_{HN} } \\ {S_{Nh} } & {S_{NH} } & {S_{NN} } \\ \end{array} } \right]$$

where *S*_*ij*_ in the matrix is derived by

17 $$S_{ij} = tr\left( {RQ_{i} RQ_{j} } \right)$$

where *tr*(·) is the trace operator, $i,j = h,H,N$, and *Q*(·) is the cofactor matrix for observables. Considering the matrix *S* may not be of full rank, the algorithm of pseudo-inverse should be used to solve Eq. (15). *R* is a matrix denoted by

18 $$R = C_{1}^{ - 1} \left[ {I - A\left( {A^{T} C_{1}^{ - 1} A} \right)^{ - 1} A^{T} C_{1}^{ - 1} } \right]$$

where *A* is an appropriate matrix corresponding to the parametric model as in Eq. (4) and *C*_1_ is the CV matrix of the observables. A CV matrix model for the heterogeneous observation types can be written as the following linear model,

19 $$C_{1} = BC_{v} B^{T} = \sigma_{h}^{2} Q_{h} + \sigma_{N}^{2} Q_{H} + \sigma_{N}^{2} Q_{N} .$$

The vector *q* can be expressed as

20 $$q = \left\{ {q_{i} } \right\},\;q_{i} = \hat{v}_{i}^{T} Q_{i}^{T} \hat{v}_{i} ,\;i = h,H,N$$

where $\hat{v}_{i} = Q_{i} Rw$, which is a vector composed of the residuals for heterogeneous observations. Substituting the formulations above into Eq. (15), we can obtain the explicit expression by

21 $$\left[ {\begin{array}{*{20}c} {tr\left( {RQ_{h} RQ_{h} } \right)} & {tr\left( {RQ_{h} RQ_{H} } \right)} & {tr\left( {RQ_{h} RQ_{N} } \right)} \\ {r\left( {RQ_{H} RQ_{h} } \right)} & {r\left( {RQ_{H} RQ_{H} } \right)} & {r\left( {RQ_{H} RQ_{N} } \right)} \\ {r\left( {RQ_{N} RQ_{h} } \right)} & {r\left( {RQ_{N} RQ_{H} } \right)} & {r\left( {RQ_{N} RQ_{N} } \right)} \\ \end{array} } \right]\left[ {\begin{array}{*{20}c} {\hat{\sigma }_{h}^{2} } \\ {\hat{\sigma }_{H}^{2} } \\ {\hat{\sigma }_{N}^{2} } \\ \end{array} } \right] = \left[ {\begin{array}{*{20}c} {w^{T} R^{T} Q_{h}^{T} Rw} \\ {w^{T} R^{T} Q_{H}^{T} Rw} \\ {w^{T} R^{T} Q_{N}^{T} Rw} \\ \end{array} } \right]$$

It is evident from the expression in Eq. (18) that an iterative process should be used because the unknown variance components $\sigma_{h}^{2}$, $\sigma_{H}^{2}$ and $\sigma_{N}^{2}$ are embedded in *C*_1_. So, the estimation for the variance components must be conducted with a convergence criterion. Here the iterative performance of MINQUE is employed to determine the variance components. In practice, a convergence criterion should be specified to terminate the computation. In this paper, the computation should not be stopped until all the values are equal.

## Numerical results and discussions

### Description of data

Here a rugged area bounded by 39°N and 41°N, and 82°W and 85°W, is chosen for testing the theory. Several numerical studies are implemented with the two datasets composed of GPS-leveling heights, gravimetric geoid data obtained from gravimetrical data and the initial cofactor matrices for ellipsoidal, orthometric heights and gravimetric geoid.

**Fig. 1:**
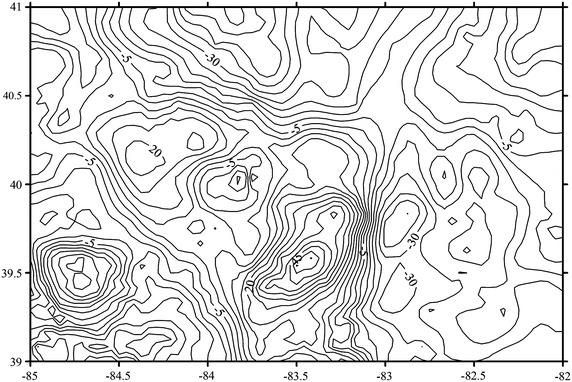
The FA gravity anomalous of tested area (unit: mGal).

A data set of Free Air (FA) gravity anomalies used for geoid computation are consisted of 4,574 ground data irregularly distributed in the test area and are referred to GRS80, and the data set of FA gravity anomalies is referred to the NAD83. All data have been removed the duplicate points and validated Least squares collocation (LSC). The error ratio used in this method is about 8 % and the remaining points are used for test (Tscherning 1991; Gil et al. 1993). Figure 1 gives the isoline map of FA gravity anomalous gridded by Kriging interpolation.

This gravity anomaly is used to estimate the geoid by application of Stokes’s formulae. Before implementing Stokes’s formula, the gravimetric geoid computations require that the topography effect should be reduced by removing step of the remove-compute-restore (RCR) technique for the purpose of making the gravity anomalous small and easy to be gridded (Guo and Xu 2011b). Here a method of Residual Terrain Model (RTM) is employed. With this technology only the topographic irregularities relative to a smooth reference surface (Omang and Forsberg 2000) are taken into account. Table 1 shows the statistical results of the FA gravity data and the residual gravity anomaly, where Δ*g*_*fa*_ is the FA anomaly, Δ*g*_*ref*_ is the effect of the reference field of EGM2008 on the gravity anomalies (Pavlis et al. 2008), Δ*g*_*rtm*_ is the terrain effect of RTM reduction with the smooth reference surface, e.g. of resolution 120 km, and Δ*g*_*res*_ is the residual gravity anomalous obtained by subtracting the effect of a global Earth gravity field model Δ*g*_*ref*_ and the RTM effect of the topography Δ*g*_*rtm*_ from Δ*g*_*fa*_, $\Delta g_{res} = \Delta g_{fa} - \Delta g_{\text{ref}} - \Delta g_{\text{rtm}}$. From Table 1, it is obvious that the reduced gravity anomaly is significantly smoother than the FA gravity anomalous.

**Table 1:** Statistics of the gravity anomalous (unit: mGal)

	Min	Max	Mean	STD
Δ*g*_*fa*_	−52.5	56.4	0.2	22.9
Δ*g*_*ref*_	−47.5	17.4	−1.8	15.6
Δ*g*_*rtm*_	−12.4	13.5	−0.6	3.7
Δ*g*_*res*_	−37.1	39.1	2.5	15.0

*Δg*_*fa*_ FA anomalies, *Δg*_*ref*_ effect of the reference field of EGM2008 on the gravity anomalies, *Δg*_*rtm*_ terrain effect of RTM reduction, *Δg*_*res*_ residual gravity anomalies.

**Fig. 2:**
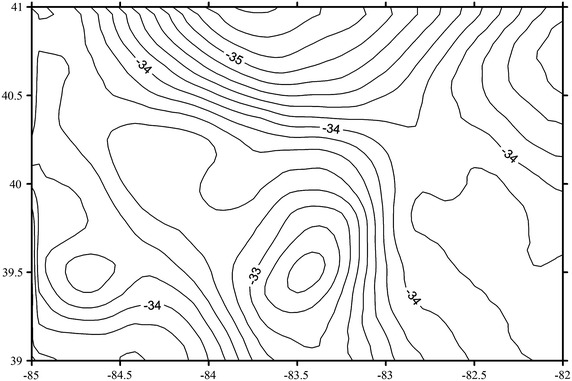
Gravimetric geoid determined with RTM model.

Then, the residual gravity anomalous are gridded on a 2.5′ × 2.5′ grid using Kriging interpolation in order to meet the requirements of geoid determination employing the technique of Fast Fourier transform (FFT) (Haagmans et al. 1993). By modifying Stokes formula as convolution integrals, the FFT techniques are used to estimate the residual geoidal undulation with the reduced gravity anomalous here. Finally, the gravimetric geoid can be obtained after applying the restoring step. The gravimetric geoid with a resolution of 2.5′ × 2.5′ is shown in Fig. 2.

**Fig. 3:**
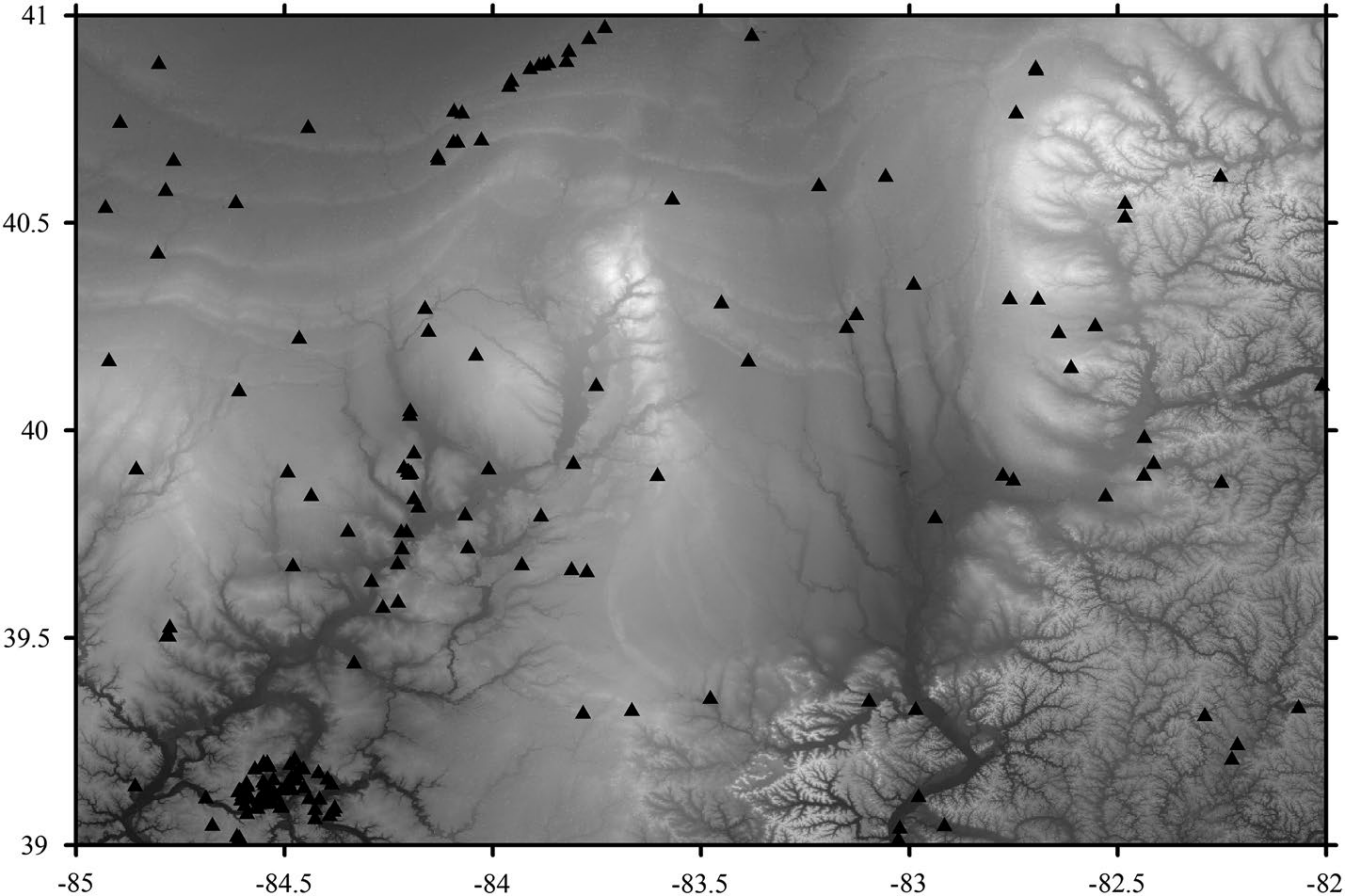
Distribution of GPS benchmarks.

The GPS-leveling heights are employed to estimate the absolute and relative accuracies of gravimetric geoid. A total number of 170 GPS-leveling benchmarks distributed throughout the test area were used. Figure 3 gives the distribution of GPS-leveling stations. The GPS observations were processed with the Bernese GPS software version 4.2 with observation periods between 4 and 16 h. All the GPS heights used in a LS adjustment are given with respect to the GRS80 reference ellipsoid, the reference frame is ITRF2005. Geodetic leveling observations are given with respect to the North American Vertical Datum of 1988 (NAVD88).

Fully populated initial CV matrices are computed for ellipsoidal, orthometric heights and gravimetric geoid data. The CV matrix for the ellipsoidal heights is obtained from the results of the GPS post-processing software package being used. These are the direct results without considering the spatial, temporal and physical correlations between GPS phase observables. Similarly, the CV matrix for the orthometric height are obtained from the rigorous adjustment of leveling measurements and the CV matrix for the gravimetric geoid heights at the GPS benchmarks are derived in a straightforward manner by applying the law of error propagation to the geoid solution. Table 2 summarizes the characteristics of the ‘a-priori’ CV matrices of all the previous data types for comparison purposes.

**Table 2:** Initial CV matrix characteristics (unit: cm)

	GPS	Leveling	Geoid
Average σ	0.7	0.6	2.0

### Iterative MINQUE scheme

When estimating variance components, convergence depends on the given initial values of weight of the measurements. Here we will study an algorithm to improve the convergence speed and behavior of estimating variance components.

Considering the availability of fully populated CV matrices is a luxury, the iterative application of MINQUE is used to estimate the variance components in practice. First of all, suppose that by using a conventional technique of variance component estimation, we have the three estimates at the *j*th step $\hat{\sigma }_{h}^{j}$, $\hat{\sigma }_{H}^{j}$, $\hat{\sigma }_{N}^{j}$. To continue the next iteration, we compute the new set of two variance components, from which, the weights for the next iteration can be further computed,

22 $$\left. \begin{gathered} P_{h}^{j + 1} = 1 \hfill \\ P_{H}^{j + 1} = \frac{{\left\{ {\hat{\sigma }_{h}^{2} } \right\}^{j} }}{{{{\left\{ {\hat{\sigma }_{H}^{2} } \right\}^{j} } \mathord{\left/ {\vphantom {{\left\{ {\hat{\sigma }_{H}^{2} } \right\}^{j} } {P_{H}^{j} }}} \right. \kern-0pt} {P_{H}^{j} }}}} \hfill \\ P_{N}^{j + 1} = \frac{{\left\{ {\hat{\sigma }_{h}^{2} } \right\}^{j} }}{{{{\left\{ {\hat{\sigma }_{N}^{2} } \right\}^{j} } \mathord{\left/ {\vphantom {{\left\{ {\hat{\sigma }_{N}^{2} } \right\}^{j} } {P_{N}^{j} }}} \right. \kern-0pt} {P_{N}^{j} }}}} \hfill \\ \end{gathered} \right\}$$

**Fig. 4:**
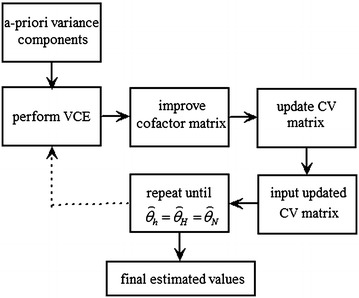
Iterative procedure of variance component estimation.

The iterative procedures are given in Fig. 4. From Fig. 4 we can see that the iterative procedures need a priori values for the estimation of variance components which have been shown in Table 2, and need to specify a convergence criterion to determine when to terminate the process. In this scheme, the process should be stopped until all the values satisfy the converge of $$\overset{\lower0.5em\hbox{$\smash{\scriptscriptstyle\frown}$}}{\theta }_{h} = \overset{\lower0.5em\hbox{$\smash{\scriptscriptstyle\frown}$}}{\theta }_{H} = \overset{\lower0.5em\hbox{$\smash{\scriptscriptstyle\frown}$}}{\theta }_{N}$$.

**Fig. 5:**
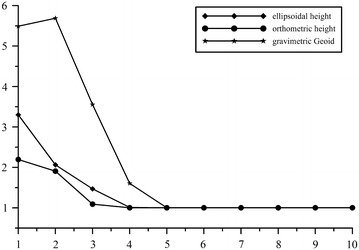
Behavior of variance-component estimated by MINQUE.

In order to study the correlations among observations, numerical tests are performed with diagonal CV matrices. Figure 5 shows the convergence behaviors of the estimated variance components by MINQUE in each of successive iterations for observable. The results demonstrate the computational efficiency.

### Numerical examples for applying MINQUE to combined LS adjustment

A popular approach is to compute the difference between the gravimetrical geoidal undulation *N*, with ellipsoidal height *h* from GPS measurement and orthometric height *H* from leveling measurement $\Delta N = h{-}H - N$. The discrepancies Δ*N* denote an evaluation for the accuracy of gravimetric geoid. In order to estimate the accuracy of gravimetric geoid, the different between gravimetric geoid solutions are compared with 165 GPS-leveling heights.

**Fig. 6:**
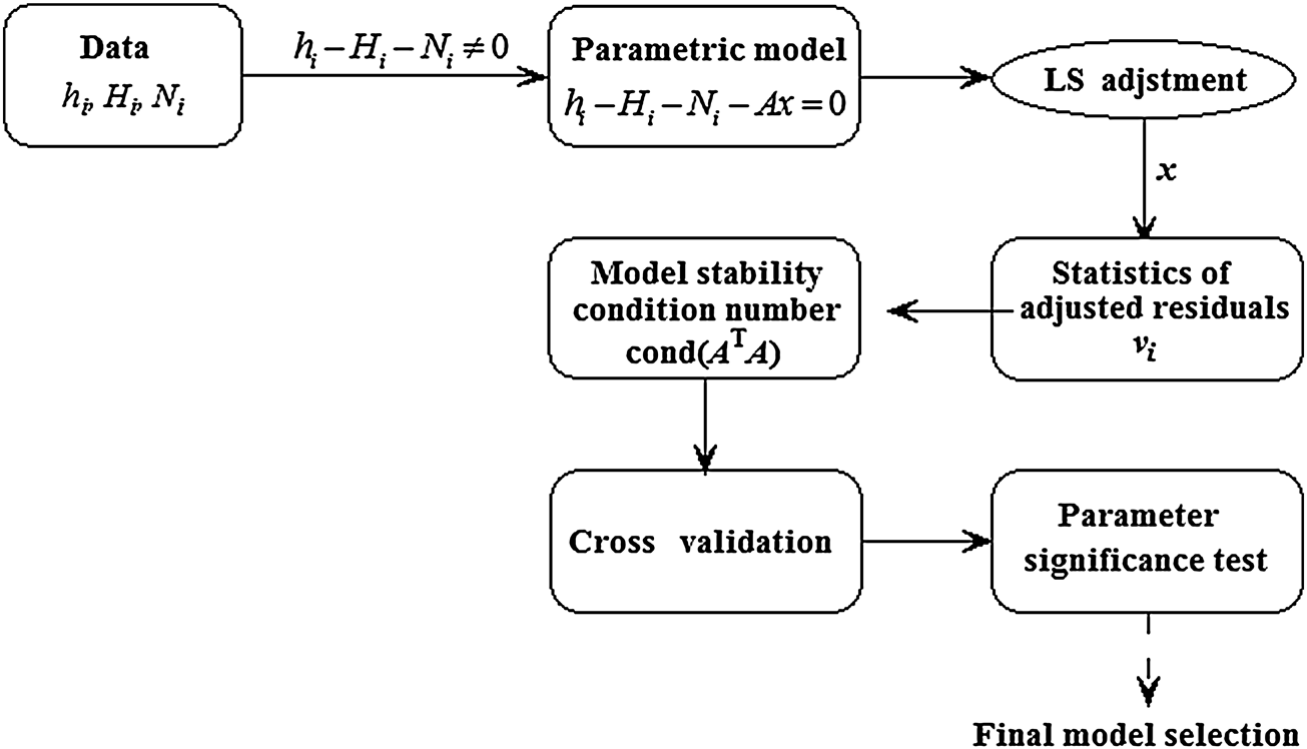
Flowchart of assessment meth for parametric model.

Each of the height data types refers to different reference surfaces which results in that biases are introduced into the geoid model. In order to absorb the errors due to the datum inconsistencies between the gravimetric geoid and GPS-leveling heights, a parametric model has been used. In general, the process of choosing the parametric model suffers from a high degree of arbitrariness. In order to evaluate the availability of parametric models, the semi-automated assessment procedure combined with F-significance test is used. Figure 6 gives the computational process of semi-automated assessment procedure. The detailed procedures are not elaborated in this paper. The interested reader should refer to the work by Dermanis and Rossikopoulos (1991). According to the results, multi-surface function is selected for computing the adjusted residuals here.

Since the main idea of this paper is to discuss the influence of a priori CV matrices on the final computed variance components, two numerical examples are conducted to assess the gravimetric geoid accuracy in this section. In the scheme of case study II, combined height adjustment is conducted by using the a priori CV matrices as given in Table 2. In the scheme of case study I, combined height adjustment is conducted by using variance-component estimated by MINQUE method. The widely used approach in case study I is the comparison of different geoid model computed by the scheme of case study II over the same region, which is an external method to evaluate the accuracy of gravimetric geoid.

Such a scheme selects the best GPS benchmarks to evaluate the accuracy of geoid with minimum range and standard deviation criteria. The selected GPS benchmarks are used to fit geoid using the multi-surface function fit technique. 150 GPS benchmarks are selected to fit geoid models in this paper. The residual values at the all the rest GPS benchmarks denote the relative accuracy of the geoid. The statistical results of the residuals at the 15 GPS benchmarks are shown in Table 3. We can see that the accuracy of geoid fitting with GPS-leveling heights based on VCE is with minimum range and standard deviation.

**Table 3:** Statistical results of the absolute and relative accuracies of gravimetric geoid (unit: cm)

Fit-model	Inside precision				Outside precision			
	Max	Min	Mean	SD	Max	Min	Mean	SD
Case study I	2.24	−1.97	0.52	1.25	8.98	−7.42	2.41	4.70
CASE study II	1.32	−0.89	0.16	0.29	5.40	−4.63	1.15	1.91

## Conclusions

The main focus point of this study is to give a procedure of geoid determined with available high precise gravimetric geoid and GPS and leveling heights and to improve the accuracy of geoid. In the context, a precise formula for the geoid computation is derived, and the relationship between ellipsoidal, orthometric heights and gravimetric geoid data is conducted. And a detailed algorithm uses the complete description of the CV matrices of the observation vector and of the coefficient matrix, possibly with unknown components of each. In the practice of this context, the iterative application of MINQUE is employed to test the variance components matrices for heterogeneous height data. Numerical case studies shows that the best results were obtained combined with the variance components estimation, with an outside precision of 1.91 cm and an inside precision of 0.29 cm, while the classical method with a-prior CV matrix is a bit worse, with an outside precision of 4.70 cm and an inside precision of 1.25 cm. The results of numerical examples show the capability and effectiveness of variance components estimation procedure in combined adjustment for calibrating geoid error model.
